# An Exploration of COVID-19 Management Policies across Nine African Countries

**DOI:** 10.24248/eahrj.v4i2.634

**Published:** 2020-11-26

**Authors:** Myron O. Odingo

**Affiliations:** Keele University, School of Pharmacy, Hornbeam Building, ST5 5BG, Newcastle Under-Lyme, Staffordshire, United Kingdom

## Abstract

**Background:**

The Coronavirus disease 2019 (COVID-19) has registered more than 16 million cases and has been declared a global pandemic. Social distancing measures have been recommended as part of health policies aimed at reducing the transmission of the disease. These have resulted in adverse social and economic implications; many countries are therefore discussing exit strategies for the relaxation of COVID-19 restrictions.

**Aim:**

To explore the COVID-19 management policies and their outcomes among 9 African countries in order to guide the upcoming and ongoing relaxation of containment and mitigation measures.

**Methods:**

Daily COVID-19 statistics were obtained from the World Health Organization between12th March 2020 and 17th July 2020). Data on government policies was obtained from the Human Data Exchange Program between 20th January 2020 and 24th July 2020, a service operated by the United Nations Office for the Coordination of Humanitarian Affairs (OCHA). Data analysis was conducted using the Python (version 3) programming language modules: Pandas, NumPy, Matplotlib, Seaborn and SciPy

**Results:**

The most common containment and mitigation measures were under the categories of; health systems strengthening, enhanced detection measures, implementation of quarantine measures, movement restrictions and social distancing. Countries with low cases and low deaths prioritised social distancing and movement restriction policies, while countries with high cases and high deaths focused on quarantines, closures of public places and borders and public communication. High cases with low death areas implemented health systems strengthening, social distancing, detection and logistics/security improvement. Low cases with high death countries focused on public communication and improved detection measures.

**Conclusion:**

The current study found that social distancing measures remain an effective method of controlling COVID-19. However, coordination between government and organisations to develop social distancing protocols within businesses and specialist organisations such as the military, prisons, educational facilities and the transport industry was observed in countries with better control of the disease.

## BACKGROUND

The Coronavirus disease 2019 (COVID-19) caused by the severe acute respiratory syndrome Corona-virus 2 (SARS-CoV2) emerged in Wuhan, China in the late 2019^1^. The disease has since been declared a global pandemic with more than 16million confirmed cases and 644,000 deaths globally^2^. Africa registered its first case of the disease in Egypt on the 14th of February, 2020^3^. It took 90 days for the continent to register 100,000 confirmed cases of COVID-19 (April-May, 2020), and the only 31 days to reach 300,000 confirmed cases (June 2020)^2^.

COVID-19 is transmitted through respiratory droplets and contaminated surfaces with potential for airborne transmission. In the absence of effective pharmacological interventions for COVID-19, the World Health organization (WHO) and the Centres for Diseases Control and Prevention (CDC) recommended social distancing as part of collective measures to reduce disease transmission. This translated into management policies that revolved around movement restrictions, health systems strengthening and public communication across Africa, and globally.^4, 5^ COVID-19 management policies have resulted in unsustainable social and economic implications including job loss and economic downturns. In Africa, unique problems surround the protection of rural population, slum dwellers and people facing humanitarian crises. Furthermore, an effective treatment for the disease may not be available until later in 2020. This combination of factors indicating prolonged disease presence has led to discussions about relaxation of COVID-19 restrictions in many countries.^6, 7^

WHO has projected that up to 44 million cases could be confirmed and up to 190,000 people could die of COVID-19 in Africa^8^ by the end of the first year of the pandemic. This prediction is based on the weaker health systems found in Africa and the large numbers of people who cannot access medical treatment. Due to this unique situation in African countries, it is important to assess the effectiveness of the current COVID-19 management policies to guide the relaxation of the restrictions currently in place.^8^

### Aim of the Study

The current study aimed to explore the COVID-19 management policies and their outcomes among 9 African countries in order to guide the upcoming and ongoing relaxation of containment and mitigation measures.

## METHODS

Daily records of COVID-19 data (number of confirmed cases and number of deaths) were obtained from the WHO website^2^ (WHO, 2020 – 12th March 2020 to 17th July 2020). Data on government actions in developing countries was obtained from the Human Data Exchange program^9^ (HDX, 2020 – last updated 24th July 2020), an online service provided by the United Nations Office for the Coordination of Humanitarian Affairs (OCHA). At the commencement of data analysis on the 24th July 2020, these databases which were aggregated by international organisations provided open access to COVID-19 statistics and control measures that were otherwise diffi-cult to access.

The control measures recorded in the HDX dataset consisted of 12 categories; Decontamination of Physical Spaces, Detection, Economic and Social Measures, Government Coordination and Legal Authorisation, Logistics/Supply Chains and Security, Movement Restrictions - At National Borders, Movement Restrictions - Within the Country, Public Communications and Education, Quarantines, Social Distancing - Closures, Social Distancing - Physical Distancing Between People, Strengthening the Healthcare System. Within these categories, there were a total of 101 specific measures to control the spread of COVID-19.

The study assessed the policies implemented in the 9 African countries listed below; Cote d’Ivoire, Ethiopia, Guinea, Kenya, Nigeria, Rwanda, Senegal, South Africa and Morocco.

Analysis was conducted using the Python (version 3) programming language modules; Pandas, NumPy, Matplotlib, Seaborn and SciPy. Ethical approval was not required since the analysis involved secondary data abstraction.

## RESULTS

South Africa registered substantially higher numbers of COVID-19 cases compared to other African countries; this was followed by Nigeria and Morocco (Table 1). The general trend was that countries with a large number of cases registered a large number of deaths, exceptions were observed in Côte d’Ivoire, Kenya and Senegal. Côte d’Ivoire reported a low number of deaths in comparison to the total number of cases in the country, while Kenya and Senegal reported a high number of deaths relative to the total confirmed cases. (Table 1)

**TABLE 1:** Summary COVID-19 Statistics

Country	New cases	New deaths	Case fatility rate	Number of measures adopted
South Africa	324221	4669	1.44	75
Nigeria	34854	769	2.21	60
Morocco	16545	263	1.59	64
Cote d'Ivoire	13403	87	0.65	53
Kenya	11673	217	1.86	46
Ethiopia	8803	150	1.7	43
Senegal	8481	156	1.84	47
Guinea	6359	39	0.61	45
Rwanda	1473	4	0.27	54

**FIGURE 1.**
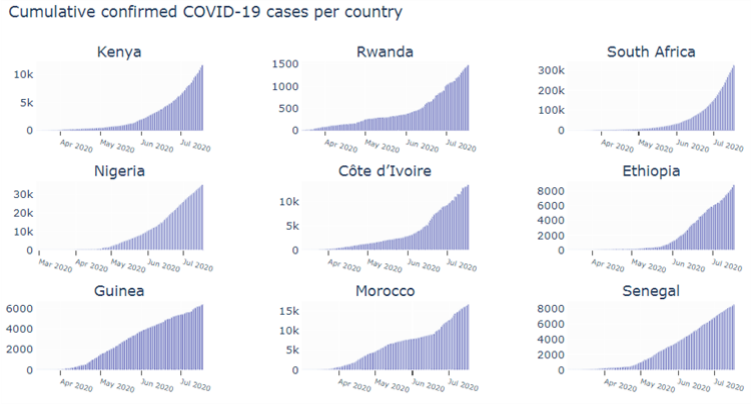
Cumulative Frequency Graph by Country

Case fatality rates (calculated as number of deaths/total number of cases) were highest in Nigeria, Kenya, Senegal and Ethiopia. Despite having a high number of cases, Morocco, South Africa and Côte d’Ivoire reported lower case fatality rates compared to other countries. Cumulative frequency graphs were drawn for each country to assess the reduction of infection cases as shown in the figure below (Figure 1). No country appeared to be ‘flattening the curve’; however, there were clear differences in the rates of infection and the total number of cases. (Figure 1)

Overall, these data indicated that the 9 African countries could be categorised in 4 ways based on the total number of cases and the case fatality rate. The high death category was classified as a case fatality rate above 1.7, while high case load was defined as cases above 13,000 by 24 July 2020.

1) High Case - Low Death (HCLD): Côte d’Ivoire, Morocco, South Africa
2) High Case – High Death (HCHD): Nigeria
3) Low Case - High Death (LCHD): Kenya, Senegal, Ethiopia
4) Low Case – Low Death (LCLD): Guinea, Rwanda

The 9 countries implemented measures from the 12 categories of containment and mitigation measures for COVID-19 noted from the HDX dataset. The categories with the highest number of actions were:Health Systems Strengthening (13), Detection Measures (11), Quarantine Activities (11), Movement Restriction (12), Social Distancing and Logistics/Supply Chain and Security Improvements (10). The countries with the largest numbers of cases implemented the largest number of actions against the 101 total measures to control the disease i.e. South Africa (75), Morocco (64) and Nigeria (60). Ethiopia implemented the least number of measures with 43. Some measures were implemented partially in some countries; Guinea (17), Ethiopia (16) and Nigeria (16) implemented the largest number of partial actions.

**FIGURE 2.**
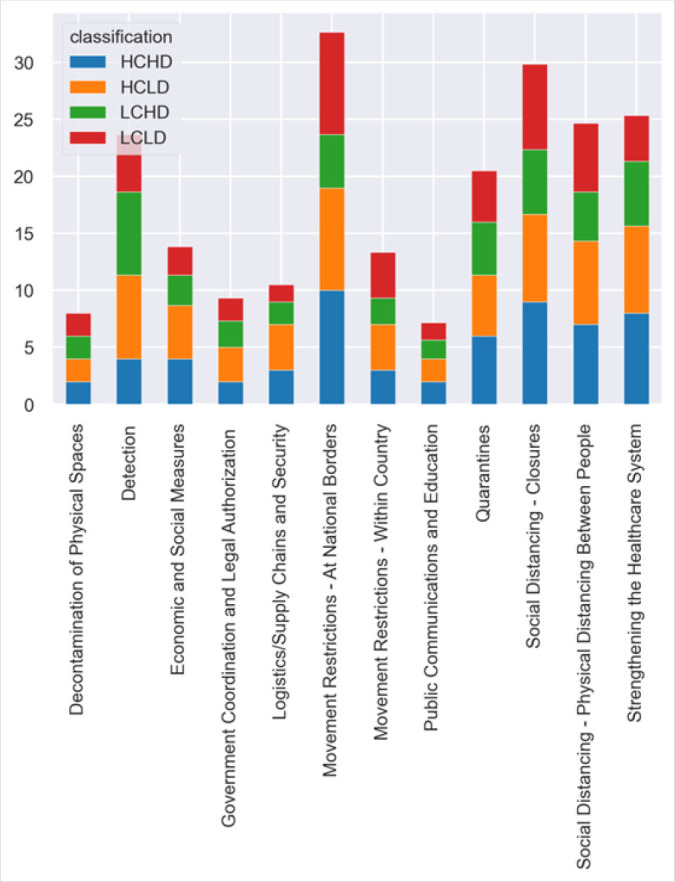
Action Taken by the Categories of Countries

The figure (Figure 2) shows on average, the measures that were adopted by the categories of countries listed above.

Countries within the HCLD category focused on social distancing (physical distancing between people), improvement of detection systems, health systems strengthening, logistics and security enhancement. HCHD countries focused on social distancing closures, quarantines, movement restriction at borders and public communication/education. LCHD countries appeared to have uneven distribution of actions with no focus on specific containment and mitigation measures. In general these countries responded by implementing government legislation, improving detection and engaging in public communication and education measures. LCLD countries focused on social distancing closures and physical distancing, movement restrictions at national borders and within the country.

## DISCUSSION

The COVID-19 pandemic has continued to spread globally and in the absence of effective pharmacological interventions. The containment and mitigation measures implemented to control the spread of the disease have resulted in adverse socioeconomic implications that are unsustainable in the long term for global economies^3^. Various exit strategies have been proposed^6^, but Africa faces a unique situation where the healthcare systems are not robust and capacity is lacking to effectively manage COVID-19.^10,11,12^

Social distancing and movement restrictions within the country were associated with lower cases and lower deaths due to COVID-19. Movement restrictions imposed by HCLD and LCLD countries appeared to be stricter than in other countries, travel was only allowed for a range of reasons and surveillance of transport systems was enforced. HCLD and LCLD countries also developed special control measures for prisons and the military. It appeared that countries with improved capacity or organisation (e.g. South Africa and Rwanda) were better placed to enforce stricter measures while supporting both healthcare providers and the general public. This was evident in the implementation of economic and social control measures where these countries provided extended medical leave for COVID-19 patients, special payments and support for those made redundant due to the pandemic and partial coverage of wages for businesses that did not lay off workers^5, 10^. In these countries the requirement to stay home was implemented as an additional control measure; however, this could be difficult to sustain because many Africans rely on day-to-day business for sustenance.

Health systems strengthening and improvement of healthcare logistics/supply chains and security were linked to a reduction in case fatality rate. However, a similar trend was observed in more resourceful countries that were able to mobilise more workers faster. Countries with better control of COVID-19 were able to deploy the military to assist with various functions in the control of the disease. Temporary hospital and care facilities were built to manage increased patient numbers, there was increased recruitment of healthcare/key workers and increased pay in some countries^9^. Overall, only countries such as South Africa and Rwanda implemented these measures consistently highlighting the inadequacy of resources. From a logistics perspective, countries with lower deaths due to COVID-19 reported coordinated planning between the government and supermarkets, pharmacies and providers of other essential services in order to ensure acquisition of these services/products while conforming to social distancing regulations.

The immediate indication is that it may be possible to develop safe methods of relaxing COVID-19 restrictions in African countries. Although African countries would struggle to strengthen health systems, many of the measures implemented in HCLD and LCLD countries could be classified as social determinants of health (hygiene, social distancing, use of masks et cetera) which could be replicated at low cost in other countries. The results indicate that a combination of physical social distancing, the use of face masks and controlled movement within the country could potentially reduce the risk of a huge outbreak. Stricter measures such as allowing travel for a limited range of reasons and the coordination between government and providers of essential services may also be necessary. Other measures such as health systems strengthening, the improvement of healthcare supply chains and continuous public communication and education remain important even though the capacity to implement them may be low.^13, 14^

Two additional measures may be critical for the successful lifting of lockdown measures in Africa. Firstly, capacity for surveillance research should be increased in order to monitor the potential for a new wave of infections. This would thereby determine whether the relaxation of containment and mitigation measures was conducted in an optimal manner. The initial slow growth of cases and low mortality/case fatality rate of COVID-19 in Africa have been attributed to reduced travel and exposure to China, climate and the presence of a younger demographic in the more populated urban centres among other reasons^15^. The younger population within urban communities in particular could mean that surveillance systems could be focused in urban settings allowing for reduced cost of implementation.

Secondly, equitable provision of healthcare is an important consideration in African countries. A majority of Africans live below the poverty line and rely on daily business activities in order to survive. These include rural populations, slum dwellers and people facing humanitarian crises^11, 15, 16^. It is therefore difficult for the African population to uphold closures and movement restriction measures^16, 17^. This could provide a potential explanation for the finding that Nigeria (HCHD) implemented closures, social distancing and health systems strengthening but maintained a High Case High Death profile. A ground roots approach involving trained community health workers may be necessary to evaluate medication access, and compliance with containment and mitigation measures especially in impoverished areas.

## CONCLUSIONS

Based on these findings, the following recommendations are provided to lessen the possible negative effects of the-lifting of lockdown rules (Table 2). These measures were adopted variably across the African countries studied but were more common in countries with good control of COVID-19. (Table 2)

**TABLE 2:** Recommendation of Control Measures

Physical social distancing in public areas and the use of masks
Restricting travel to a limited number of reasons
Coordination between government and common businesses to uphold control measures e.g. pharmacies, restaurants
Development of specifc control measures for the military, prisons and educational facilities
Establishment of temporary health facilities to reduce overcrowding
Surveillance of covid-19 especially in urban areas to assess
Training of community health workers to ensure adherence to control measures at the local level

However, direct enforcement of these measures through coordination between government and common businesses and organisations produced better control of the disease. This coordination involved development of specific protocols within businesses and specialist organisations such as the military, prisons, educational facilities and the transport industry.

### Limitations

COVID-19 statistics are largely driven by the rate of testing within countries. In the absence of complete and detailed data including patient and disease it is difficult to make projections or to suggest specific control policies. Furthermore, with different policy-making and resource mobilisation capacities between countries it is difficult to judge the extent of implementation of the policies within the HDX dataset. These factors effectively hinder more comprehensive statistical analyses. Nevertheless, currently available data have to be used in order to guide decision making. The present study bypasses these challenges by adopting a simple exploratory or observation based methodology that does not require comprehensive data. The number and type of control measures adopted in each country in relation to the resulting cases of infections and deaths are used to provide a rationale for lifting disease control restrictions.
